# Healthy participant engagement in early clinical trials: results from the European EUFEMED survey

**DOI:** 10.3389/fphar.2025.1540948

**Published:** 2025-08-01

**Authors:** J. Klein, T. van Iersel, D. de Vries, Y. Donazzolo, I. Klingmann

**Affiliations:** ^1^ SGS, Clinical Pharmacology Unit, Antwerpen, Belgium; ^2^ Clinical Pharmacology, ICON, Groningen, Netherlands; ^3^ Clinical Research and Development, Miltenyi, Leiden, Netherlands; ^4^ Eurofins Optimed, Grenoble, France; ^5^ Pharmaplex, Wezembeek-Oppem, Belgium

**Keywords:** healthy volunteer, participant centricity, early clinical development, layman’s review, participant engagement

## Abstract

**Introduction:**

While numerous healthy volunteers contribute to clinical trials on a yearly basis, the aspect of including these participants in the drug development process, like the patient-centric approaches, are not well known. To gain broader insights in the aspect of healthy participant engagement, preferences, and motivation, we performed a European wide survey. Additionally, a literature search on the topic of healthy participant engagement was performed.

**Methods:**

An online questionnaire containing 61 questions on demographics, motivation, informed consent, engagement, transparency, and preferences was created, combining five-point Likert and open text field answers. The questionnaire was translated in several European languages and shared among early phase clinical trial units within Europe. Additionally, a literature search was performed on healthy participant engagement.

**Results:**

A total of 4,349 completed questionnaires were eligible for analysis. Countries with adequate number of responses were Belgium, France, Netherlands, Germany, Hungary and United Kingdom. Altruistic motivation was the primary reason for participation. The Informed Consent Form (ICF) paragraphs on risk, schedule of assessments and restriction was better read than the financial aspects, ethics and data protection, with variations between gender, age, experience and country. Only 71.2% of the responders finds an ICF written in correct lay language and 44.6% is willing to assist in ICF review on adequate lay language. In the literature search, no articles described healthy participant engagement.

**Discussion:**

The benefit of including patients in the drug development process has been proven in multiple publications and is a movement that is being advocated more frequently. Healthy participant engagement is not known yet, while similar benefits can be suggested. As altruistic reasons are the main motivation for participation, engaging participant in the clinical trials might enhance their motivation. Together with all stakeholders, description of methods for healthy participant engagement should be initiated to increase willingness to contribute to clinical trials.

## 1 Introduction

Involving patients in the drug development process and clinical trial design has become more common in recent years. The concept of patient-centricity is described in literature, as well as in guidelines in ICH-E8(R1) (International Council for Harmonisation of Technical Requirements for Pharmaceuticals for Human Use, 2021) which were regionally translated by the FDA (FDA, 2024) and EMA (European Medicines Agency, 2022), and the draft version of ICH E6 (R3) (International Council for Harmonisation of Technical Requirements for Pharmaceuticals for Human Use, 2023). By involving patients in creating development plans, the needs, concerns, and real-world experiences of these patients are considered. This also increases patient recruitment (Crocker et al., 2018), patient treatment compliance, regulatory approval, and results in fewer substantial modifications (Stergiopoulos et al., 2020). Patient engagement encompasses education and information to patients, co-creation, access, and transparency (Yeoman et al., 2017). However, patients show different levels of interest and report different reasons for getting involved, much dependent on their level of knowledge about clinical trials, the seriousness of their disease and the existing treatment alternatives (Schilling et al., 2019).

Most early phase trials are performed on healthy volunteers. These early phase clinical trials require a highly standardized health profile and recruitment occurs proactively by the clinical trial site, often from an existing volunteer database, thus reducing the clinical trial recruitment timelines. Endpoints regarding safety and tolerability, pharmacokinetics and pharmacodynamics can be largely determined in healthy participants. However, this population has no health-related benefit from participating in clinical trials. Despite the critical role of healthy participants in medicines development, no guidelines, or scientific articles present considerations on the option of engaging healthy volunteers into different aspects of the trials in which they participate.

Engaging healthy volunteers differs from patient engagement as they do not have indication experience or a need for access to a new treatment for themselves or their patient community. Their role in advising the sponsor on study design, patient-relevant endpoints or relevant comparator within development plans is therefore limited. However, they have an interest in risks and burden of the clinical trial in which they participate, and in the information they receive. Therefore, better understanding should be developed on healthy participants’ expectations and preferences for education, information, and transparency as well as in their interest in co-creation as described by Yeoman for patient centricity (Yeoman et al., 2017).

As the basis for a European discussion on needs, pros and cons of healthy participant involvement and preferences in early phase clinical trials, the European Federation for Exploratory Medicines Development (EUFEMED) has launched a survey targeting participants in healthy volunteer trials over the European continent. This survey included questions on healthy volunteer engagement and adequate education from clinical trial operators. Additionally, a literature review was performed to investigate implementation regarding healthy volunteer participation. The literature review and survey are used complementary to create a broad overview on the topic. To our knowledge, this is the first publication on multi-country opinion collection from early phase participants about their level of interest and motivation in engagement in trial design, condition development, and interest in receiving trial results.

## 2 Materials and methods

A survey questioning the motivation, needs, preferences, and insights of participants in early phase healthy volunteer trials was created. It contained seven demographic questions, six on motivation for participation in clinical trials, 14 related to Informed Consent, Healthy Participant Engagement, and transparency, 16 on overall preferences and needs and 18 on preferences and needs during admission to a clinical trial site. These questions were based the Maslow’s theory on motivation (Van Iersel, 2022) and the experience of the authors. The answers to the demographic questions were listed based on the type of question (example for demographics, see Table 1). The other questions had a five-point Likert scale, where responders needed to choose “strongly disagree,” “disagree,” “neither agree nor disagree” “agree,” “strongly agree.” The Likert scale is commonly used for psychometric objective and is validated as such (Joshi, et al., 2015). The collected data from the Likert scale is deemed to be ordinal of nature. Every section was concluded with a comment and elaboration free text field section.

**TABLE 1:** Survey questions and answers related to demographics.

Question	Answers
What is your age?	18–25 years/26–35 years/36–45 years/46–65 years/66 years or over
What is your gender?	Female/Male
In which country do you live?	Dropdown list of all European countries
What is your daily occupation?	Working full time/Working part time/Student/Not working or retired
In how many clinical studies have you participated up to this day?	None/1/2–4/5–9/10 or more
Have you participated in a clinical trial as a healthy volunteer and/or as a patient?	Healthy volunteer/Patient/Both
In which countries did you participate in a clinical study?	Dropdown list of all European countries

The survey was shared with early phase clinical trial centers in Europe with translation into local languages. The local translations were performed by native speakers, addressed by the working group. The available languages were English, Dutch, German, French, Greek, Hungarian, Danish, Spanish, and Polish based on the full translations that have been received. The translations were set up in JotForm (Jotform.inc., San Francisco, CA, United States) and tested by the working group before publication. The link to the online JotForm survey was sent to early phase clinical trial centers in Europe with a request to share the link with their healthy volunteer database. The GDPR policy of EUFEMED was followed and no personal data on the responder nor the site that shared the link was collected. Anonymous responses were collected from 25 July 2023 until 13 November 2023.

After closing the survey, the dataset was cleaned in Excel. Double entries were removed, and personal or clinical site data added by responders in the free text fields was redacted using three asterixis. The cleaned dataset was entered into IBM SPSS Statistics 29.0.2.0 for statistical analysis. In this analysis, only descriptive statistics was used.

PubMed was used to evaluate publications on ((“Healthy participant”) OR (“healthy subject”) OR (“healthy volunteer”)) AND ((engagement) OR (empowerment) OR (enhancement)) on 16 January 2024, after finalization of the survey. All titles were reviewed to determine relatedness to the objective. The publications deemed related to the objective were fully read to review descriptions of healthy participant engagement.

## 3 Results

### 3.1 Literature review

A total of 437 publications were identified in the PubMed database. Upon reviewing the titles, only three articles were deemed related to the objective. One article described a specific approach to healthy participant engagement (Walker et al., 2022). Walker et al. (2022) emphasized the importance of inviting and incentivizing healthy volunteers to contribute to the improvement of phase I trials through community engagement or other mechanisms. They focused on sharing their experiences and perspectives in the clinical trials they participated in, as well as how competent authorities and independent review boards could facilitate this. No suggestions were made to create involvement in clinical trial activities.

The remaining two articles did not specifically address healthy participant engagement. The first article, authored by Fisher and Walker (2019), discussed a phase I framework aimed at identifying gaps in ethics and the policy framework surrounding clinical trials. The second article focused on the Volunteers in Research and Ethics (VolREthics) initiative, which is an international community discussing topics related to the protection of healthy volunteers in clinical trials such as participant registries to prevent over-volunteering, fair compensation, and avoidance of exploitation (Fisher, 2023).

### 3.2 Survey results

**FIGURE 1:**
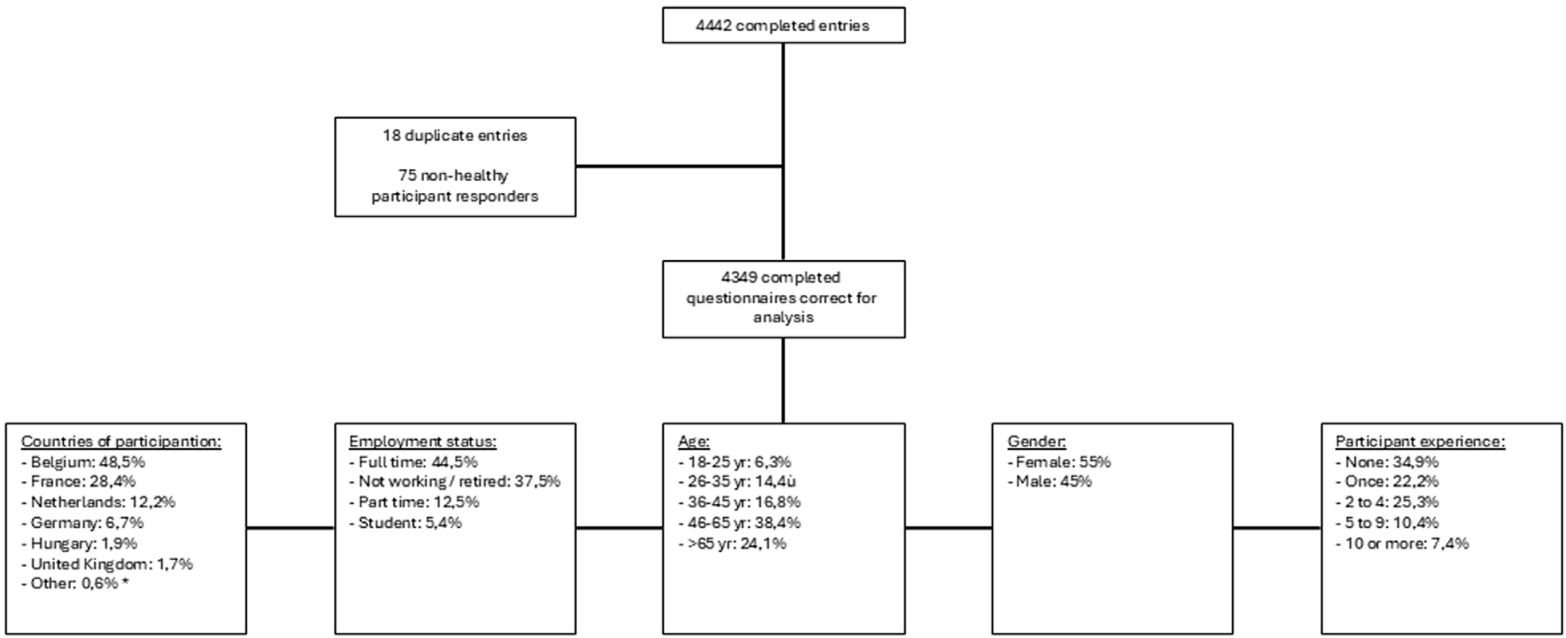
Results of questionnaire entries. (*) several countries had one to three entries: Albania, Austria, Bosnia and Herzegovina, Bulgaria, Croatia, Czech Republic, Estonia, Finland, Greece, Italy, Latvia, Lithuania, Poland, Portugal, Romania, Serbia and Spain.

A total of 4,349 completed evaluable questionnaires were available for analysis. The analysis was descriptive, no statistical analysis was performed. The demographics, countries of origin, employment status and participation experience are shown in Figure 1 below.

The primary motivation for participating in clinical trials were altruistic reasons, selected by 47.9% of the respondents, while 41.0% cited monetary motivation as their primary reason.

Most respondents indicated that they carefully read all sections of the Informed Consent Form (ICF). The percentage of respondents who answered “Strongly Agree” or “Agree” to the question whether they carefully read the potential risks section in the ICF was 88.0%. For the indication and mechanism of action this percentage was 84.5%, for the schedule of assessments 88.0%, for restrictions during the trial 87.3%, for the financial aspects 80.4% and for the ethical considerations, protection of personal data and insurance it was 72.0%. Female respondents showed slightly higher levels of careful reading across all sections of the ICF compared to male respondents. There were no major differences observed among different age groups. However, younger respondents tended to read the restrictions during the trial section more carefully (92.4%) compared to respondents aged 66 years or older (84.3%). Similarly, younger respondents also paid closer attention to the financial aspects section (89.1%) compared to the eldest population (65.6%). The oldest age group, on the other hand, was more interested in the ethical aspects and data protection (75.3%) in comparison to the 26- to 35-year-old respondents (63.2%). Respondents from the United Kingdom and Germany were more focused on the schedule of assessments (91.9% and 91.7% respectively) compared to those from the Netherlands (85.9%). Hungarian respondents showed more interest in reading the financial aspects section (93.9%) than the Belgian (76.7%). French respondents focused more on the ethical considerations section (77.7%) compared to the respondents from Belgium (69.4%). Students were the most attentive to the restrictions section (92.0%), while showing less interest in the ethical considerations and protection of personal data section (64.1%). Conversely, individuals who were not employed or retired demonstrated the least interest in the sections pertaining to the financial aspects (72.3%). Among respondents who previously had participated in 10 or more clinical trials there was a high response of “strongly agree” and “agree” on carefully reading the financial aspects section (86.0%).

A 71.2% of the respondents found that the ICF was presented in well-understandable lay language. Reviewing the ICF by an experienced participant for correct language was considered a good idea by 59.3% of the respondents, and 44.6% confirmed that they would be willing to participation in the ICF review. Additionally, 43.5% expressed interest in following training to become a reviewer, with 42.5% of the respondents expecting a fee for this review. No major differences in such contribution were found in gender and working status. The age groups of 26–35 and 36–45 years showed the highest interest in participating in a review (47.1% and 48.8% respectively). Hungarian respondents expressed the highest agreement in that ICFs were presented in well-understandable lay language (92.7%), while German respondents were the least in agreement (64.4%) and at the same time were the most skeptical of having the ICF reviewed by an experienced participant (44.2%). United Kingdom participants demonstrated the highest motivation to contribute to such a review (59.5%). The more trials respondents had participated in, the more they found the level of lay language terms sufficient. Participants who had not yet participated in any clinical trials expressed the highest agreement that a review might be useful (65.7%). The participants who had the most extensive experience in clinical trials (10 or more participations) showed the highest interest in contributing to the ICF review, with 41.8% expressing their willingness. Similarly, within the group of participants who had participated in five to nine trials, 37.0% also expressed their interest in contributing to the review.

When it came to transparency, 91.0% of the respondents expressed a desire to receive the results of their health check (screening results) and 89.9% stated that they would like to receive the lay summary of the results. No major differences were observed here between gender, age, working status and clinical trial experience. Hungarian respondents showed the highest interest in receiving their health check results (96.3%), while the Belgian respondents were most interested in the lay summary (92.3%).

## 4 Discussion

Patient centricity is a widely recognized and established concept in the field of clinical research. The concrete involvement of patients in different steps of the clinical development process (Hoos et al., 2015), in ethical review of clinical trials by ethics committees (Klingmann et al., 2018) as well as in benefit-risk assessments in the marketing authorization process and scientific advice (Murphy et al., 2022) by competent authorities aims at increasing the relevance of new therapeutic options for patients living with that disease, at improving the protection of the trial participants and at ultimately increasing the trust in new medicines. Pharmaceutical companies are seeking input from patient organizations during the drug development process, with the aim of incorporating patient preferences into their plans. This includes organizing, e.g., focus groups to discuss specific protocols and involving patients in trial design decisions (Farah et al., 2023). The revision three of the ICH E6 (GCP) guideline, together with the revised ICH-E8 guideline, are now specifically emphasizing the importance of considering inputs from various stakeholders, including healthcare professionals and especially patients, on the clinical trial aspects like trial objectives, design and activities as well as content of informed consent materials and any other participant-facing information (International Council for Harmonisation of Technical Requirements for Pharmaceuticals for Human Use, 2023).

While the benefits of patient engagement in the planning, preparation and reporting of clinical trials are widely recognized, there is hardly any discussion about the effects of engaging healthy volunteers in Phase 1 trial activities in addition to their role as trial participants, reflected by a lack of literature on that topic. However, it is worth noting that experienced participants, including healthy volunteers, can provide valuable insights that can benefit sponsors and investigators. The benefit for this engagement can be expected to include both the healthy participants as the industry.

Comparable to feedback from patients interested in engaging with sponsors and investigators in improvement of clinical trials (Chakradhar, S, 2015; Parsons et al., 2015; IQVIA, 2022), also the healthy volunteers in this survey expressed interest in getting access to education in clinical trials methodology. The human pharmacology community should therefore elaborate and share ideas and experiences in how best to educate healthy participants, e.g., in study-related information sessions, local community teaching opportunities, dedicated online sessions or through social media options. Priority should be given to include them in the informed consent and the lay summary of results creation as these were seen as most interesting areas for contributions by the survey respondents. To stimulate opportunities for healthy participant involvement but also to protect their rights and give advice, healthy participants could be encouraged and assisted in forming a healthy participant organization that offers information and education to healthy people wanting to contribute to clinical research in whatever capacity.

According to our survey and against the wide-spread opinion of clinical researchers, the main motivation for the responding healthy participants to contribute to clinical trials was rooted in altruism rather than monetary gain. Participants feel a sense of purpose in helping to develop future therapeutics, particularly if they have a personal connection to someone affected by a specific disease. However, when age is taken into consideration, responders from 18 till 45 years of age had the stipend as main motivation. Given the overall predominance of altruistic motivation, it is expected that these participants would like to know if their contribution was indeed useful. In accordance with the European Union Clinical Trial Regulation, sponsors are required to submit a lay summary of the clinical trial results to the EU Clinical Trial Information System (CTIS), which can be accessed by the general public in the CTIS Public Portal. Such a summary is highly desired by nearly 90% of survey respondents and should therefore be a key consideration for sponsors. However, for the summary to be effective, it must be easily accessible and written in plain language that can be understood by all readers. Since the target audience for the lay summary is healthy participants, involving a clinical trial participant in reviewing the draft can greatly improve its content presentation and readability. This is supported by the EU Guidance on Good Lay Summary Practice (European Commission Directorate-General for Health and Food safety, 2021) which defines the same conditions for lay summary planning, development, translation and dissemination reporting results from all types of clinical trials.

The Informed Consent Form (ICF) is the most important document for trial participants. Its primary purpose is to explain the trial rationale, objectives, risks, benefits, burden, and obligations when joining and thus to enable the participant’s informed decision to participate. From a legal standpoint there is a tendency to view the ICF as a contractresulting in overly formal and legal language and a very lengthy ICF. This tendency impairs the original objective of the informed consent process in enabling the participant “to make an understanding and enlightened decision”, as stated in the Nuremberg Code in 1949 (Permissible Medical Experiments, 1949) which principles were incorporated by the Declaration of Helsinki in 1964. The objective of the ICF as concise and focused presentation of key information of the clinical trial was reiterated in similar wordings in 1979 in the Belmont report (National Commission for the Protection of Human Subjects of Biomedical and Behavioral Research, 2024), in 1997 in ICH E6 (GCP), in the United States in the 2017 Common Rule (Fed Regist, 2017), as well as in the Regulation EU 536/2014 (“Clinical Trial Regulation”). While most participants carefully read all the sections of the ICF before signing, 28.8% of survey respondents disagreed with the statement that the ICF was written in clear and concise language. This is causing concerns, as the ICF plays a crucial role in ensuring participants’ voluntary and informed consent is in line with the principles outlined in the Declaration of Helsinki. The majority of respondents stated that having an experienced participant review of the ICF would add value to the process. Professionals who work on these topics full-time may struggle to gauge the common knowledge and understanding of non-experts, often resorting to using scientific language when communicating with participants. Healthy participants, who do not have medical or scientifical backgrounds, can read and identify phrases in the ICF that are difficult to understand, and offer suggestions for improvement. Approximately 44.6% of the respondents were motivated to help in such a review, indicating both a need and motivation to enhance communication between healthy participants and researchers. This finding was consistent across different age groups, genders, levels of clinical trial experience and employment status.

Independent Ethics Committees are mandated in Europe to give advice to the investigator and sponsor concerning the ethical aspects of the trial and to review documents that are presented to participants. The primary focus of these committees is to ensure the protection of the safety and wellbeing of the participants. While reviewing the documents, the use of suitable plain language emerges as an important topic, often giving rise to questions and potential concerns. One potential strategy to streamline the review process and expedite the timeline from submission to approval of a clinical trial is to include healthy participants with experience in clinical trials in the content development and unexperienced participants in testing its readability (International Council for Harmonisation of Technical Requirements for Pharmaceuticals for Human Use, 2021).

Reviewing the responders’ demographics revealed that almost half of the responders were from Belgium, creating an overrepresentation of responses from this country. Nonetheless, there were sufficient responders (at least 75) from each of 6 European countries in which larger Clinical Pharmacology Units are located (Belgium (48.5%), France (28.4%), Netherlands (12.2%), Germany (6.7%), Hungary (1.9%), United Kingdom (1.7%)). Differences per country are described in the results. When interpreting the overall results, it needs to be considered that the overrepresentation from Belgium, with a majorly of responders in age groups 46–65 years and full time employed, can create an imbalance. A large portion of 34.9% of the responders has not participated in a trial yet. As this is the first study objectifying the need for healthy participation, this imbalance was expected. No stratification was performed to guarantee equal distribution on demographics. In further research, the findings of this article with the current limitation can be used as a guidance for a more statistically powered conclusion. Due to the variation in several variables, only descriptive analysis could be performed and no statistical significancy was planned to be calculated. Further evaluation regarding country specific variables needs to be performed. For example, it can be suggested that the willingness to participate for the health check-up is more relevant in eastern-European countries with a higher threshold for first-line medical support in comparison to western-European countries. The impact of the review of the document within the Clinical Trial Regulation might impact the results in comparison to the review in the United Kingdom. Several countries have template Informed Consent Forms, or work with supplementary part in the ICF to reduce the number of pages.

The literature researched focused on healthy participant engagement or empowerment. It was found that there were no relevant articles describing this concept, nonetheless information can be found on specific concepts related to healthy participant engagement. To boost recruitment and retention is a longer follow-up trial, the HEALTHY trial conducted a post-pilot study to understand the reason for participation and implemented these results for the trial itself (Drews et al., 2009). The motivation for participation (Fisher, 2018) and the characteristics of early phase participants (Corey et al., 2021) have been described and need to be used as a cornerstone to further develop healthy participant engagement.

Despite the lack of existing literature on healthy participant engagement, this article presents findings directly from the community itself. Respondents recognized the benefits of increased engagement and expressed a willingness to contribute. Obviously, a larger than expected portion of participants is motivated to support the clinical researchers in the clinical trial development process. Further research and joined discussions should occur to develop a methodology for constructively engaging healthy participants in this process to mutual benefit. Lessons learned from the involvement of patients in therapeutic trials can help to speed-up efficiency gains.

Suggestions for implement healthy participant engagement can be based on the concept of patient engagement (Table 2). Yeoman et al. (2017) described that the patient engagement consists of four criteria: Education and Information, Cocreation, Access and Transparency. Using this blueprint, several methods of implementation can be suggested. The review of draft Informed Consent Forms part of Cocreation and review of the Laymen’s summery assists on transparency of clinical trial results. Proactively sharing screening results gives participants insight in their general health and wellbeing. Including healthy participants in information sessions or community visits can enhance their engagement towards the clinical trials.

**TABLE 2:** The criteria for patient engagement described by Yeomen ea. compared to suggestions for Healthy Participants Engagement.

Criteria	Patient engagement	Healthy participant engagement
Education and information	• Patient advocates and patient stories	• Educate other healthy participants through experience
	• Helping patients manage their own health and make their own decisions	• Assist on reading scientific language
	• Resources for families, carers, and communities	• Educate the local community to improve diversity in the clinical trial
	• Resources for healthcare professionals	• Teaching how to report effects of the treatment correctly
	• Collect patient views and provide information about side effects	
Cocreation	• Work with patients and other stakeholders throughout the research, development and launch of medicines	• Input in design, restrictions and days hospitalized
	• Research patient’s wider needs	• Creation on the informed consent form to make it easy understandable
	• Work with patients and other stakeholders to advocate policies in the interests of patients	
	• Group of stakeholders to codesign solutions	
Access	• Support service to help patients navigate complex health systems and issues	• Support other healthy participants in finding suitable clinical trials
	• Patient assistance programme	• Creation of healthy participant organizations to assist
	• Flexible pricing policy	
Transparency	• Transparency with clinical trial data	• Review of the layman’s summary on correct plain language
	• When developing medicines, report on the measurable patient benefit and patient safety	• Access to screening data
	• Respond to patients’ feedback-show how you have listened	• Building a partnership and bilateral trust
	• Open and accountable reporting of progress	• Value feedback for improvement and adequate response
	• Values-based approach to business development	• Guidance in finding published information on the clinical trial participated

Together with all stakeholders, the healthy participants, sponsors, regulatory agencies, ethics committees and investigators, the possibility to include healthy participants in the early phase clinical trial process should be initiated as described in this article. A working party with these stakeholders and organizations like the VolRethics initiative should be created to draft a guideline on how to implement the concept op healthy participant engagement. Freely available educational tools like the European Patient Academy on Therapeutic Innovation (EUPATI) should be used to increase the knowledge of interested healthy participants to collaborate with clinical researchers on improving subject protection, trial suitability, and result reporting from early phase clinical trials.

## 5 Conclusion

Healthy clinical trial participants play a crucial role in the advancement of medicines development pathways. While much literature exists on patient engagement in clinical trials, there is a notable lack of information regarding the involvement of healthy participants. Considering this gap, this survey was initiated to investigate - and the results indicate - the potential benefits of fostering engagement of healthy volunteers in early phase research. The healthy volunteer community recognizes the necessity of this engagement and expresses a willingness to contribute towards this endeavor. Therefore, it is urged that this mandate be embraced, prompting further discussions to establish effective systems and evaluate the associated benefits.

## Data Availability

The raw data supporting the conclusion of this article will be made available by the authors, without undue reservation.
